# Trial Protocol: A randomised controlled trial of extended anticoagulation treatment versus routine anticoagulation treatment for the prevention of recurrent VTE and post thrombotic syndrome in patients being treated for a first episode of unprovoked VTE (The ExACT Study)

**DOI:** 10.1186/1471-2261-13-16

**Published:** 2013-03-09

**Authors:** Jayne Tullett, Ellen Murray, Linda Nichols, Roger Holder, Will Lester, Peter Rose, FD Richard Hobbs, David Fitzmaurice

**Affiliations:** 1 Primary Care Clinical Sciences, School of Health and Population Sciences, University of Birmingham, Edgbaston, Birmingham B15 2TT, UK; 2 Haemophilia Unit, Queen Elizabeth Hospital, Birmingham, B15 2TH, UK; 3 Warwick Hospital, Lakin Road, Warwick, CV34 5BW, UK; 4 Department of Primary Care Health Sciences, University of Oxford, New Radcliffe House, Walton Street, Oxford, OX2 6NW, UK

**Keywords:** Venous thromboembolism, Deep vein thrombosis, Pulmonary embolism, Extended warfarin, Post thrombotic syndrome, D-dimer

## Abstract

**Background:**

Venous thromboembolism comprising pulmonary embolism and deep vein thrombosis is a common condition with an incidence of approximately 1 per 1,000 per annum causing both mortality and serious morbidity. The principal aim of treatment of a venous thromboembolism with heparin and warfarin is to prevent extension or recurrence of clot. However, the recurrence rate following a deep vein thrombosis remains approximately 10% per annum following treatment cessation irrespective of the duration of anticoagulation therapy. Patients with raised D-dimer levels after discontinuing oral anticoagulation treatment have also been shown to be at high risk of recurrence.

Post thrombotic syndrome is a complication of a deep vein thrombosis which can lead to chronic venous insufficiency and ulceration. It has a cumulative incidence after 2 years of around 25% and it has been suggested that extended oral anticoagulation should be investigated as a possible preventative measure.

**Methods/design:**

Patients with a first idiopathic venous thromboembolism will be recruited through anticoagulation clinics and randomly allocated to either continuing or discontinuing warfarin treatment for a further 2 years and followed up on a six monthly basis. At each visit D-dimer levels will be measured using a Roche Cobas h 232 POC device. In addition a venous sample will be taken for laboratory D-dimer analysis at the end of the study. Patients will be examined for signs and symptoms of PTS using the Villalta scale and complete VEINES and EQ5D quality of life questionnaires.

**Discussion:**

The primary aim of the study is to investigate whether extending oral anticoagulation treatment (prior to discontinuing treatment) beyond 3–6 months for patients with a first unprovoked proximal deep vein thrombosis or pulmonary embolism prevents recurrence. The study will also determine the role of extending anticoagulation for patients with elevated D-dimer levels prior to discontinuing treatment and identify the potential of D-dimer point of care testing for identification of high risk patients within a primary care setting.

**Trial registration:**

ISRCTN73819751

## Background

Venous thromboembolism (VTE) is associated with significant mortality and morbidity, including post-thrombotic syndrome (PTS). VTE is considered as a chronic disease with pulmonary embolism (PE) and deep vein thrombosis (DVT) being different manifestations of the same disease process.

Currently, patients with a first episode of unprovoked proximal DVT or PE, with no apparent other risk factors for recurrent VTE, are given a course of oral anticoagulation (warfarin in the UK) treatment which is then discontinued. The optimum duration of warfarin therapy depends on the individual patient and has not been defined [1]. Concern in terms of duration of treatment is due to the risk of bleeding from prolonged warfarin treatment. Several studies have investigated the optimal duration of anticoagulant therapy with the British Committee for Standards in Haematology (BCSH, 1998) recommending between six weeks and six months, depending upon the aetiology [2,3].

A substantial proportion of patients – up to 17% [4-7] go on to have a recurrent episode of VTE which can be a potentially life-threatening condition [8]. If recurrence occurs, the patient is normally managed thereafter with long-term warfarin, provided there are no contraindications. Although optimal duration of anticoagulation treatment for patients with a first idiopathic VTE remains uncertain, randomised trials have suggested that patients should be treated for a minimum of three months [9] but beyond that the duration of anticoagulation has little effect on the rate of recurrence^3^. There is an annual recurrence rate following a first VTE of approximately 10% per annum following treatment cessation, irrespective of the duration of warfarin therapy [10]. This suggests that some patients with a first unprovoked VTE should continue warfarin in the long-term however we are currently unable to identify this population. Studies of extended treatment with low dose warfarin have confirmed that maximal benefit is conferred at standard therapeutic targets rather than fixed low dose regimes [11,12].

It would be very useful to have an accurate predictor of recurrence of VTE to decide which patients with a first episode of unprovoked proximal DVT or PE would be most likely to benefit from long term oral anticoagulation, and also which patients should only receive short term therapy.

D-dimer is a fibrin degradation product, present in the blood after a blood clot is degraded by fibrinolysis and levels of D-dimer can be tested using laboratory and point-of-care (POC) testing devices. The primary clinical use of D-dimer testing has been in conjunction with probability scores to determine whether further investigation is required for the diagnosis of VTE. More recently interest has been shown in studies which have investigated whether D-dimer can be utilised as a guide for determining who is at risk of recurrent VTE following treatment of the initial acute episode [13,14].

Previous small studies outside the UK have suggested that D-dimer levels could act as a useful predictor of recurrent thromboembolism in patients whose warfarin is discontinued [14-16]. There are limited data on utility of the D-dimer as a predictor in patients still on therapy, but Rodgers *et al.* have shown that D-dimer can be used to predict recurrence in low risk female patients [17]. In addition to D-dimer levels a recent meta-analysis has suggested that age and gender may be effective in predicting recurrent VTE [18]. This study concluded that men are at greater risk of developing a recurrent VTE after a first unprovoked episode and that age could be used as predictor in women. Therefore D-dimer levels could be a potentially useful clinical tool to decide which patients, with a first episode of unprovoked proximal DVT or PE, would most likely benefit from long-term warfarin therapy.

The conclusions of 2 systematic reviews of D-dimer testing after stopping treatment for VTE, (7 studies with a total of 1888 patients) were: “additional research is needed to establish the optimal interval between stopping anticoagulation and performing D-dimer testing, to identify the optimum cut-off that predicts recurrence and to develop a clinical prediction rule for recurrent DVT.” [19], and “strategies which incorporate a number of high performing baseline and post-baseline predictors could be more effective in predicting recurrent VTE and should be tested. If oral anticoagulation is continued, there is no evidence on the duration of this extended therapy [20].”

The only UK data included in these reviews were from a prospective cohort study of 272 patients with a first episode of venous thrombosis that was unprovoked or provoked by a non-surgical trigger. They undertook D-dimer testing 1 month after cessation of warfarin therapy and found a non-significant difference in recurrence (5.5/100 patients years in D-dimer + ve *vs.* 4.1/100 patient years in D-dimer –ve) [10].

PTS is a frequent and costly complication of DVT which can lead to chronic venous insufficiency and ulceration. PTS has a cumulative incidence after 2 years of around 25% and it has been postulated that prolonged treatment with oral anticoagulants could prevent the development of PTS [21]. The only data available from a randomised trial suggested no association between long-term low dose warfarin treatment and development of PTS [22]. The prevalence of PTS in this study was 37% after 2.2 years, with severe PTS in 4%. A recent study demonstrated an association between D-dimer level and the development of PTS [23], this is something that will be investigated during the course of this study.

## Methods

### Aims and objectives

Considering patients with a first unprovoked proximal DVT and PE who have been treated with 3–6 months oral anticoagulation, the aims of the study are:

•To investigate the effect of extending treatment with oral anticoagulation in terms of reduction in incidence of VTE and PTS

•To develop an algorithm to predict high/low recurrence rates in these patients

As part of this we will be investigating the following:

•To establish the performance characteristics of D-dimer testing whilst still on treatment as a prediction tool for recurrence of VTE in patients with first unprovoked proximal DVT or PE.

•To establish factors which contribute to the recurrence of VTE in patients with a first unprovoked proximal DVT or PE.

•To determine the cost-effectiveness of the measurement of D-dimer and subsequent extended treatment with anticoagulation.

•To determine patient preferences and utilities with regard to extended warfarin treatment.

### Participants

Patients will be recruited through a number of primary care and secondary care anticoagulation clinics in the wider Midlands area over a two year period.

Patients identified as receiving warfarin for a first unprovoked proximal DVT or PE and who are eligible for the trial (Table 1) will receive a brief information sheet about the study during their anticoagulant clinic appointment. They will also be given a post card to return to the research department stating their willingness in principle to take part in the study, for their GP to be contacted and their records to be accessed for the purpose of checking their eligibility for the study. The patients GP will simultaneously be sent a summary of the trial and informed that one of their patients wishes to participate. The GP will be asked to complete a form to state whether they wish their practice to take part in the study.

**Table 1:** Study inclusion and exclusion criteria


**Inclusion criteria**	People aged 18 years or over with a diagnosis of first unprovoked^*^ proximal^** ^DVT or PE on treatment with anticoagulants
	Have completed 3 – 6 months of anticoagulant therapy (target 2–3)
	^*^The episode will be defined as unprovoked as long as there is no history within the previous 3 months of:
	major surgery; lower limb trauma e.g. fracture, cast, limping for 3 days; use of the combined oral contraceptive pill or hormone replacement therapy ; pregnancy; significant immobility e.g. confined to bed for 3 days; active cancer.
	^**^Proximal refers to a DVT alone in the trifurcation area, popliteal, superficial femoral, deep femoral, common femoral and iliac veins.
**Exclusion criteria**	Patients under the age of 18 years
	Patients with another indication for long term warfarin therapy e.g. Atrial Fibrillation
	Patients with a diagnosis of first unprovoked proximal DVT or PE who are no longer on anticoagulation therapy
	Patients with a high risk of bleeding as evidenced by any of the following:
	• Patients with a previous episode of major bleeding where the cause was not effectively treated
	• Known thrombocytopaenia with a platelet count of less than 120 ×10^9^ /L
	• Known chronic renal failure with a creatinine of more than 150 μmols/L (1.7 mg/dl) or eGFR less than 30
	• Known chronic liver disease with a total bilirubin of more than 25μmols/L (1.5 mg/dl)
	• Active peptic ulcer
	• Patients requiring antiplatelet therapy (eg Aspirin and/or Clopidogrel)
	Patients with a vena cava filter in place
	Patients who have had a D–dimer test performed within 2 months of potential enrolment other than for evaluation for suspected recurrent VTE that was not confirmed
	Patients whose GP expects their life expectancy to be less than 5 years
	Patients unable to attend follow up visits due to geographic inaccessibility
	Patients participating in a competing investigation
	Patients with known antiphospholipid syndrome
	If patients are subsequently found to have antiphospholipid syndrome then they will have to be excluded.
	Patients with known inherited protein C and/or protein S or antithrombin deficiency
	If patients are subsequently found to have protein C and/or protein S or anithrombin deficiency then they will have to be excluded.
	Patients with a diagnosis of active cancer
	Patients unwilling to give consent

Those patients who reply with a positive response from willing general practices will be contacted to determine that they still wish to take part in the study. If still willing to take part then the research nurse or a member of the practice staff will access the patients’ primary care notes to assess their eligibility for inclusion into the study. If the patient is eligible then they will be followed up (at least 1 month before the end of their current treatment) to arrange their first visit to the GP surgery. Any additional travel costs and prescription costs will be paid to the patient from study funds.

Those with a negative reply will not be contacted. Those patients who do not return their postcard will be given a second postcard a month after the first one was given out. If a patient wishes to take part in the study but their GP is unwilling, alternative arrangements will be made at a hospital clinic.

The first appointment at the GP clinic will inform as to the nature of the trial. For those patients willing to participate the research nurse will obtain written informed consent.

### Trial intervention

Study participants will have a total of six visits over a two year period.

#### Visit 1

This is the same visit as the information, consent and randomisation visit and will be within 2 weeks of current anticoagulation treatment end date. Patients will be randomised electronically into one of two groups; Group O will discontinue warfarin after their usual clinical intervention, Group W will continue warfarin for a further two years.

Patients will be interviewed to collect the following data:

Concomitant medications; ethnicity; smoking status; alcohol consumption; medical history; BMI; and family history of VTE

A heparinised venous sample of blood will be taken for a D-dimer test on a POC device (Cobas h 232, Roche diagnostics). In addition citrated venous samples will be collected for D-dimer testing. These will be sent to a central laboratory to be frozen and stored for analysis during and at the end of the study.

The D-dimer result will be recorded centrally onto a database for future analysis and both patient and researcher will be blinded as to the result of this test.

All patients will be clinically examined for signs and symptoms of PTS using a validated clinical scoring system (the Villalta scale) and will also complete the VEINES quality of life score [24] as well as EQ 5D which allows the measurement of broad aspects of quality of life [25]. In addition the patients will complete a costs questionnaire relating to the expenses incurred on a recent anticoagulation clinic visit. Where questionnaires are incomplete or unclear the patients will be contacted by telephone to clarify and complete the responses.

Compression stockings will be used by patients as advised by the clinician treating the DVT and the researchers will not influence their utilisation.

The anticoagulation clinic that the patient would normally attend will be informed that the patient is entering a study as will any specific hospital consultants who are involved with the patients’ care (e.g. haematologists). Patients randomly allocated to Group W will be followed-up through their usual oral anticoagulation service provided in terms of warfarin management and the anticoagulation clinic lead will be asked to extend their clinic visits for a further 2 years. Patients randomly allocated at this point to Group O will undergo a further D-dimer test 1 month after cessation of treatment. Again both researcher and patient will be blinded to this result.

#### Visits 2–6

All patients randomised (Groups W and Group O) will be reviewed every 6 months for 2 years in total to assess evidence of VTE recurrence, clinical assessment of PTS, and measurement of D-dimer (again patient and researcher are blinded to these results).

All patients will again be asked to complete the VEINES-QOL/Sym a validated disease specific, quality of life score, and EQ 5D, questionnaires.

If a patient in Group O (off warfarin) has their warfarin restarted by their GP during the study period for a VTE recurrence then they will be removed from the study but a case review will be carried out in patient’s primary care notes looking for evidence of thrombotic events. If a patient in Group O has to restart warfarin for another reason e.g. atrial fibrillation, then they will continue to be followed up in the usual manner. If a patient in Group W (on warfarin) has to permanently stop taking their warfarin during the study then they will be followed up and included in the final analysis on an intention to treat basis. If a patient in Group W has a temporary break in their treatment for any reason, they will continue to follow the same visit regime, making a note of the temporary break.

Patients in Group W will be asked to return for a final D-dimer test 1 month after discontinuing warfarin.

Patients randomised into group W will continue with their warfarin management as before. INR data will be collected from the anticoagulation database at six monthly intervals throughout the study to assess overall control.

### Outcome measures

#### Primary outcomes

**Figure 1:**
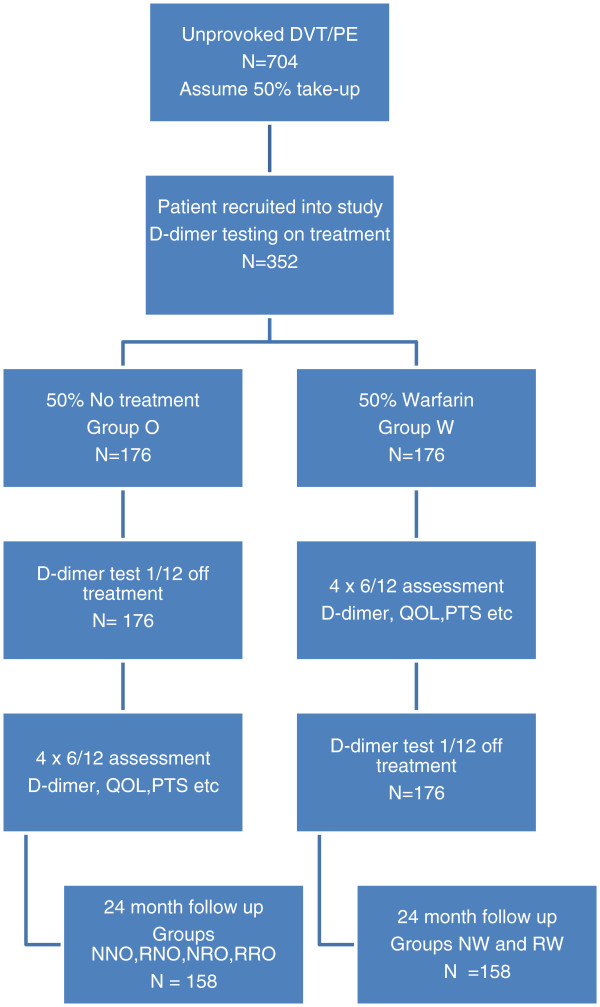
Flow chart for trial intervention.

1. Number of recurrent thrombotic events between those with raised D-dimer at point of randomisation who were randomly allocated to the treatment arm (group RW) and those with raised D-dimer at point of randomisation who were randomly allocated to the no treatment arm and produced a normal D-dimer off treatment (NRO) and those with raised D-dimer at point of randomisation who were randomly allocated to the no treatment arm and then produced a raised D-dimer off treatment (RRO) (see Figure 1)

2. Number of recurrent thrombotic events between those showing an elevated D-dimer off treatment (RO) versus those showing a negative D-dimer off treatment (NO) (see Figure 1)

#### Secondary outcomes

1. Severity of PTS between groups

2. Bleeding events - defined as in Table 2

3. INR control in terms of percentage time in range

4. Optimal cut off on a D-dimer result

5. Costs of D-dimer and subsequent extended treatment with anticoagulation

6. Cost effectiveness

7. Information on the types of patient who potentially benefit from extended warfarin treatment, age and gender

8. Patient quality of life

**Table 2:** Definition of bleeding events


**Major bleeding**	Clinically overt bleeding that is associated with:
	A fall in haemoglobin of 2 g/dl or more, or
	A transfusion of 2 or more units of packed red blood cells or whole blood, or
	A critical site: intracranial, intraspinal, intraocular, pericardial, intra-articular, intramuscular with compartment syndrome, retroperitoneal, or
	A fatal outcome
**Non-major clinically relevant bleeding**	Non-major clinically relevant bleeding is defined as overt bleeding not meeting the criteria for major bleeding but associated with medical intervention, unscheduled contact (visit or telephone call) with a clinician, (temporary) cessation of warfarin treatment, or associated with discomfort for the subject such as pain or impairment of activities of daily life.
	Examples of non-major clinically relevant bleeding are:
	• Epistaxis if it lasts for more than 5 minutes, if it is repetitive (i.e., 2 or more episodes of true bleeding, i.e., not spots on a handkerchief, within 24 hours), or leads to an intervention (packing, electrocautery, etc.) and no admission to hospital.
	• Gingival bleeding if it occurs spontaneously (i.e., unrelated to tooth brushing or eating), or if it lasts for more than 5 minutes
	• Haematuria if it is macroscopic, and either spontaneous or lasts for more than 24 hours after instrumentation (e.g., catheter placement or surgery) of the urogenital tract
	• Macroscopic gastrointestinal haemorrhage: at least 1 episode of melena or hematemesis, if clinically apparent
	• Rectal blood loss, if more than a few spots
	• Haemoptysis, if more than a few speckles in the sputum, or
	• Intramuscular hematoma
	• Subcutaneous hematoma if the size is larger than 25 cm^2^ or larger than 100 cm^2^ if provoked

### Ascertainment of outcomes

#### D-dimer results

All baseline and six monthly D-dimer results will be recorded onto the trial database by practice staff.

#### Thrombotic events and associated deaths

• At six monthly intervals and at the end of the study patient GP records will be reviewed for any mention of hospital admission, to ascertain any thrombotic events including fatalities, and whether they are still taking warfarin.

• When hospitalisation or death is identified in the study population, a discharge summary and a copy of the death certificate (if applicable) will be obtained. (The patients’ NHS numbers will be flagged at the Medical Research Information Service to inform on cause of death).

• These will be scrutinised by the Independent Adverse Event committee who will be blinded to the treatment allocation. The committee will decide what was the primary cause of hospitalisation/death. This committee will meet once all the data has been collected.

• Research nurses will abstract other information concerning the hospital admission, including admission and discharge dates, and discharge destination. Date of death will be recorded.

Recurrence will need to be objectively documented (i.e. defined radiologically). Radiological criteria for recurrent DVT will be: a new non-compressible venous segment compared to the examination at baseline; an increase of 4 mm or more in thrombus diameter with compression; or, a convincing extension in length.

#### INR control

Patients INR results, with the amount of warfarin taken, will be collected onto the trial database at 6 months, 18 months and at the end of the study.

#### Patient quality of life

This will be assessed by all patients completing questionnaires at each study visit.

Veines-QOL/Sym a validated disease specific quality of life questionnaire will be used as well as EQ-5D allows changes in health status to be measured but also valued, using the University of York Measurement & Valuation of Health general population survey tariff. Where questionnaires are incomplete or unclear the patients will be contacted by telephone to clarify and complete the responses.

#### Incidence and severity of PTS

Measured using a validated clinical scoring system (the Villalta scale). Signs will be measured on both legs [26].

#### Cost data

The cost analysis will adopt a broad perspective to include costs incurred within the health sector and by patients. Health sector costs will include additional costs of D-dimer testing and prolonged treatment (anticoagulation) costs. Patient level data will be collected on treatment and condition-specific health service utilisation (primary and secondary care), thrombotic episodes, PTS and bleeding events. Information on costs incurred by patients when attending anticoagulation clinics (e.g. time and travel costs) will also be collected with a patient cost questionnaire. Information on resource use will be collected from patient notes. Unit costs will be obtained from standard sources and health care providers.

### Sample size

Initially, patients will be randomly assigned to warfarin (Group W) or no treatment (Group O) arms Following the flow chart for analysis (Figure 1) will result in 6 study populations, those with normal D-dimer at point of randomisation who were randomly allocated to the warfarin treatment arm (group NW), those with elevated D-dimer at point of randomisation who were randomly allocated to the treatment arm (group RW), those with normal D-dimer at point of randomisation who were randomly allocated to the no treatment arm and then produced a normal D-dimer off treatment (NNO), those with normal D-dimer at point of randomisation who were randomly allocated to the no treatment arm and then produced an elevated D-dimer off treatment (RNO), those with elevated D-dimer at point of randomisation who were randomly allocated to the no treatment arm and produced a normal D-dimer off treatment (NRO) and those with elevated D-dimer at point of randomisation who were randomly allocated to the no treatment arm and then produced an elevated D-dimer off treatment (RRO). (Thus in the two or three letter code the last letter indicates the arm a patient is randomised to, the previous letter indicates the outcome of the D- dimer test performed on the same visit as randomisation and, in the case of those randomised to no further treatment, the first letter relates to the D-dimer result produced after randomisation). A 10% loss to follow up rate is assumed from the beginning of the trial. The Data Safety Monitoring Board will inform the research team if recruitment is below target as the study progresses and if so further hospitals will be recruited.

Total sample size will be sufficiently large to show a significant difference in recurrence rates between those showing a positive D-dimer on treatment who receive warfarin (RW) and those showing a positive D-dimer who receive no treatment (RNO plus RRO). Following Palareti [7] this means a sample size for each group of 79 in order to give 90% power to a 5% significance test when comparing 2 year recurrence rates of 21.4% and 4.3%. Assuming a 50% positive D-dimer rate this implies a total of 79 × 2 × 2/0.9 = 352 patients taking part in the study (and 316 completing the study). Allowing a 50% participation rate this means that 704 patients need to be approached initially. With such a sample size an overall comparison of recurrence rates between the arms W and O can be made with power of between 86 and 99% assuming recurrence rates of between 1.4% and 4.3% for the warfarin arm (Group W, depending on what recurrence rate patients with normal D-dimer on warfarin produce) and 14.2% for the No treatment arm.

One of the objectives of the study is to determine an optimal cut off on a D-dimer result taken when the patient is receiving treatment. In a study of 272 patients, Baglin *et al.* report that 170/272 patients had a D-dimer in excess of 500 ng mL-1 and their ROC analysis suggests an optimal cut off of 500 ng/ml [10]. The circumstances of their study differ from ours but using their data will give some indication of the precision with which we will be able to estimate an optimal cut off with slightly more patients than Baglin *et al.* With some assumptions, a rough calculation of the precision with which a cut off in the region of 500 could be estimated with our sample size of 316 would be within about 7% (95% confidence interval).

### Randomisation

Randomisation will be performed within the computerised clinical case report form (CRF) produced in Primary Care Clinical Sciences, University of Birmingham. When a patient is identified as being eligible for the study and has given written consent to take part, the research nurse will enter details into the computerised CRF and will be issued immediately with a dedicated randomisation code allocating the patient to either continuing warfarin therapy or stopping warfarin therapy.

Patients will be randomly allocated at this point to either continue warfarin (Group W) for a further 2 years or to discontinue treatment (Group O). Randomisation will be stratified by DVT and PE to ensure there are equal numbers in both groups W and O.

### Analysis and statistical methods

Intention to treat analysis will be used. The types of analysis proposed are Chi-square, generalized linear models, logistic regression and survival analysis, t-tests, ANOVA and non-parametric equivalents.

Analysis will be carried out at the end of the study, as there is likely to be insufficient power for interim analyses. Quality control of data collection will be conducted by a trial management committee throughout the study and by a statistician independent of the research team.

Logistic regression would be used to relate D-dimer result and other patient data to events such as recurrence, bleeding episodes and PTS separately on the two arms (Group W and Group O) of the trial and together. Baglin *et al*[10], with their 272 patients, showed a significant association between age and D- dimer (p < 0.001) and our total sample, allowing for loss to follow up, of 316 should be ample to incorporate such a relationship. In comparing D-dimer levels between genders they achieved a significance level of p = 0.18 with 272 individuals. A rough calculation would suggest a p value of 0. 15 for the same level of difference in D-dimer (approximately 80 ng/ml) in our total sample of 316 patients.

#### Cost effectiveness analysis

The analysis will determine the cost-effectiveness of the measurement of D-dimer and subsequent extended treatment with anticoagulation. Data collection will be for all trial patients so that a stochastic cost analysis can be undertaken. A cost-consequence analysis will initially be reported, describing all the important results relating to costs and consequences. Subsequently, a cost-effectiveness analysis will be conducted using data on the clinical events of interest (thrombotic and bleeding events). An incremental analysis will be undertaken to calculate the cost per major outcome averted. A cost-utility analysis will also be undertaken using patient responses to the EQ-5D questionnaire, to calculate the cost per additional Quality-Adjusted Life Year (QALY) gained.

The analysis will be from an NHS perspective, however patient costs will be incorporated in a sensitivity analysis in order to consider cost-effectiveness from a broader perspective. The data for costs is likely to have a skewed distribution therefore the plan is to explore the nature of the distribution of costs. If the data is not normally distributed, the non parametric comparison of means (e.g. bootstrapping) will be undertaken. Efforts will be made to minimise the problem of missing data, but if this does pose a problem at the economic analysis stage then imputation techniques will be used.

In addition to the trial-based economic evaluation, longer term costs and consequences will be explored by extrapolating beyond the end of the trial. Decision modelling will be undertaken using data from the trial and from non-trial sources, in order to determine the long-term cost-effectiveness of the intervention, with results reported as cost per additional QALY gained.

The robustness of the results will be explored using sensitivity analysis. This will explore uncertainties in the trial based data itself, the methods employed to analyse the data and the generalisability of the results to other settings. Uncertainty in the confidence to be placed on the results of the economic analysis will be explored by estimating cost effectiveness acceptability curves. These plot the probability that the intervention is cost effective against threshold values for cost effectiveness.

#### Data validation

Data cleaning will take place by a series of logical checks on the electronic data using specific queries. Discrepant data entries will be checked with the source documents and the database amended if necessary.

#### Data management, protection and confidentiality

The data management is fully compliant with the Data Protection Act and ICH GCP. The trial is registered with Legal Services at the University of Birmingham who are responsible for ensuring that the purposes for which the University processes personal data is registered with the Information Commissioners office.

Submissions will be made to the NHS IC Medical Research Information Service for flagging deaths, exits from and re entries to the NHS, including PCT data.

### Time plan

It is anticipated that patient recruitment will take place over a 2 year period. Patient follow-up will then continue for a further 2 years.

### Assessment of safety

Participants safety will be ensured by screening for eligibility using a structured form completed by trial healthcare professionals. This will record evidence of eligibility and the presence/absence of any inclusion and exclusion criteria. The Research Associates and Nurses will take a general medical and drug history to assess for other complicating diseases and medications. Any queries will be discussed with the trial nurse, chief investigator and the relevant physicians at the trial site. The eligibility will be signed off by the physician providing routine medical care.

### Adverse event reporting

Data will be collected for all serious adverse events (SAE), non-serious adverse reactions (NSAR), serious adverse reactions (SAR) and SUSAR as defined in Table 3. Adverse events (AEs), SAEs and suspected unexpected serious adverse reactions (SUSARs) will be reported in compliance with International Conference on Harmonisation (ICH)- Good Clinical Practice (GCP) Guidelines.

**Table 3:** Definition of adverse events

	**A fatal outcome**
**Definition of serious adverse reaction**	Any thrombotic event e.g. stroke, TIA
	Major Bleeding- Defined as clinically overt bleeding that is associated with:
	• A fall in haemoglobin of 2 g/dl or more, or
	• A transfusion of 2 or more units of packed red blood cells or whole blood, or
	• A critical site: intracranial, intraspinal, intraocular, pericardial, intra-articular, intramuscular with compartment syndrome, retroperitoneal, or
	• A fatal outcome
**Definition of a non serious adverse reaction**	Defined as overt bleeding not meeting the criteria for major bleeding but associated with medical intervention, unscheduled contact (visit or telephone call) with a clinician, (temporary) cessation of warfarin treatment, or associated with discomfort for the subject such as pain or impairment of activities of daily life.
	Examples of non-major clinically relevant bleeding are:
	• Epistaxis if it lasts for more than 5 minutes, if it is repetitive (i.e., 2 or more episodes of true bleeding, i.e., not spots on a handkerchief, within 24 hours), or leads to an intervention (packing, electrocautery, etc.) and no admission to hospital.
	• Gingival bleeding if it occurs spontaneously (i.e., unrelated to tooth brushing or eating), or if it lasts for more than 5 minutes
	• Haematuria if it is macroscopic, and either spontaneous or lasts for more than 24 hours after instrumentation (e.g., catheter placement or surgery) of the urogenital tract
	• Macroscopic gastrointestinal haemorrhage: at least 1 episode of melena or hematemesis, if clinically apparent
	• Rectal blood loss, if more than a few spots
	• Haemoptysis, if more than a few speckles in the sputum, or
	• Intramuscular hematoma
	• Subcutaneous hematoma if the size is larger than 25 cm^2^ or larger than 100 cm^2^ if provoked
**Definition of serious adverse event**	A serious adverse event is defined as an untoward medical occurrence in a participant of a study that does not necessarily have a causal relationship with the treatment that results in:
	• Death,
	• Is life threatening
	• Requires hospitalisation or prolongation of existing hospitalisation
	• Results in persistent or significant disability or incapacity
	• Consists of a congenital abnormality or birth defect
	Other known rare adverse reaction listed in the SmPC are:
	Skin reactions: Purpura and ecchymosis
	Pruritic lesions
	Skin necrosis
	Alopecia
	Diarrhoea
	An unexplained fall in haematocrit
	Jaundice and hepatic dysfunction
	Fever, nausea, vomiting and pancreatitis
	Hypersensitivity reactions are extremely rare and if any of these reactions occur they will be reported and it will be recommended to the clinician responsible for the participants warfarin management that warfarin is replaced by an alternative anticoagulant drug
	A thrombotic event can also be defined as a serious adverse event related to warfarin.

### Ethics and research governance

The trial will be conducted in compliance with the principles of the Declaration of Helsinki (1996), the principles of the ICH-GCP and run in accordance with EU Clinical Trials Directive and all of the applicable regulatory requirements. The study protocol and other documentation have been approved by Trent Research Ethics Committee and MHRA.

### Publication

The trial results will be written up for submission to a peer reviewed journal and the trial is registered with ISRCTN. No data relating to individuals will be identified in these publications.

## Discussion

This study will primarily investigate the role of extending treatment with oral anticoagulation for those patients with raised D-dimer levels prior to discontinuing treatment and will study the impact of this management on both recurrence of thrombosis and development of post-thrombotic syndrome, both extremely costly outcomes for the NHS. This strategy would allow those people who require extended anticoagulation treatment to receive it whilst ensuring that those who only require short term treatment are not exposed to unnecessary and potentially dangerous treatment.

Identifying the potential for utilisation of D-dimer POC testing will make feasible the identification of those high risk patients within a primary care setting negating the necessity for costly and inconvenient referral elsewhere. Thus people with a first unprovoked proximal DVT or PE could be managed differently to current management with the potential for significant improvements in health outcomes and patient well being.

## Abbreviations

VTE: Venous thromboembolism; DVT: Deep vein thrombosis; PE: Pulmonary embolism; PTS: Post thrombotic syndrome; NW: Randomised to Group W with normal D-dimer at point of randomisation; RW: Randomised to Group W with elevated D-dimer at point of randomisation; NRO: Randomised to Group O with elevated D-dimer at point of randomisation and then produced a normal D-dimer off treatment; RRO: Randomised to Group O with elevated D-dimer at point of randomisation and then produced an elevated D-dimer off treatment; NNO: Randomised to Group O with a normal D-dimer at point of randomisation and then produced a normal D-dimer off treatment; RNO: Randomised to Group O with normal D-dimer at point of randomisation and then produced an elevated D-dimer off treatment.

## Competing interests

None of the authors have any competing interests arising from this research.

## Authors’ contributions

DF, EM, FDRH, PR, and WL were responsible for identifying the research question. DF and EM were responsible for drafting the initial version of the protocol. DF, EM and JT have contributed to the development of the protocol. RH and LN were responsible for designing the analysis plan. JT was responsible for drafting this paper but all authors have provided comments on the drafts and have approved the final version.

## Funding

This report is independent research funded by the National Institute for Health Research under its Programme Grants for Applied Research programme (RP-PG-0608-10073: Improving the prevention and treatment of Venous Thromboembolism in Hospital and the Community). The views expressed in this publication are those of the author(s) and not necessarily those of the NHS, the National Institute for Health Research or the Department of Health.

## Pre-publication history

The pre-publication history for this paper can be accessed here:

http://www.biomedcentral.com/1471-2261/13/16/prepub
